# Recurrent networks expect

**DOI:** 10.1186/1471-2202-16-S1-P285

**Published:** 2015-12-18

**Authors:** Adam Ponzi, Jeffery R Wickens

**Affiliations:** 1 Okinawa Institute of Science and Technology Graduate University (OIST), Onna, Okinawa 904 0495, Japan

## 

How the brain anticipates future events is one of the most intriguing questions in neural information processing. The brain does not simply respond to the external world but in predictably structured environments like natural language, birdsong and somatosensory flow in fluids predicts it [1]. Reactions are improved by predictive encoding of what sensory stimuli may occur as well as when they may occur. Quantitative studies of implicit timing in streaming perceptual discrimination tasks show that performance is enhanced if stimuli occur at expected times according to an established rhythm. The neural basis for implicit timing is not fully understood but oscillatory entrainment mechanisms have been suggested [2]. Recent studies confirm that low-frequency brain oscillations do become phase entrained in such tasks. Phase entrainment increases with the temporal regularity of the sensory stream and correlates with enhanced discrimination performance [3]. It is not limited to sensory cortices; larger scale cortical networks, as well as subcortical networks like the basal ganglia [4], may also be coherently modulated by the predictability of stimuli streams. The presence of large scale oscillatory entrainment suggests that network mediated neuronal ensemble dynamics may be involved. Recurrent neural networks generate complex but reproducible temporally extended dynamical activity patterns in response to input stimuli [5,6]. Such transient activity patterns have been suggested to provide a natural substrate for working memory and a representation of elapsed time.

**Figure 1:**
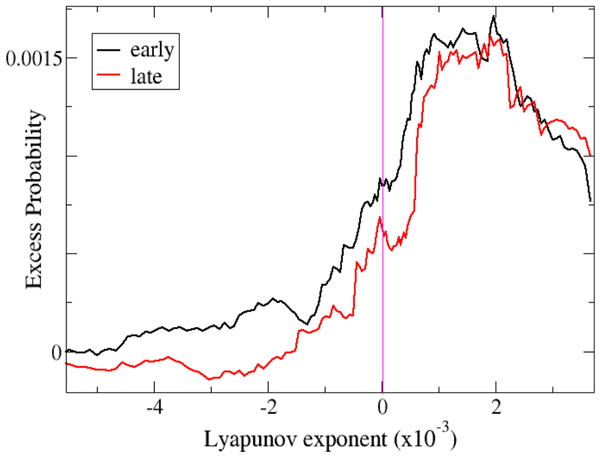
**Excess correct classification probability of on-time target stimuli compared to mistimed target stimuli (100 ms early, black ; 100 ms late, red) in a temporally regular streaming task with inter-trial-interval 800 ms**. Results are plotted versus the Lyapunov exponent of the autonomous network dynamics.

Here we add to the understanding of how random recurrent neural networks support neural information processing by demonstrating that temporal expectation also naturally emerges from their dynamics. We show that the weakly chaotic oscillations generated by recurrent networks can be phase synchronized [7] by temporally regular stimulus sequences. We show network responses are maximally discriminative when stimuli fall at their preferred phase in phase entrained networks (Figure 1). Discriminability increases continuously with both temporal regularity and stimulus type predictability which also interact. Our results do not depend on specific network characteristics, are resilient to the presence of network noise and random distractor stimuli and are consistent with multiple streaming perceptual discrimination studies.
